# Influence of Trabecular Bone on Peri-Implant Stress and Strain Based on Micro-CT Finite Element Modeling of Beagle Dog

**DOI:** 10.1155/2016/3926941

**Published:** 2016-06-14

**Authors:** Sheng-hui Liao, Xing-hao Zhu, Jing Xie, Vikesh Kumar Sohodeb, Xi Ding

**Affiliations:** ^1^School of Information Science and Engineering, Central South University, Changsha, China; ^2^Department of Stomatology, The First Affiliated Hospital of Wenzhou Medical University, Wenzhou 325000, China

## Abstract

The objective of this investigation is to analyze the influence of trabecular microstructure modeling on the biomechanical distribution of the implant-bone interface. Two three-dimensional finite element mandible models, one with trabecular microstructure (a refined model) and one with macrostructure (a simplified model), were built. The values of equivalent stress at the implant-bone interface in the refined model increased compared with those of the simplified model and strain on the contrary. The distributions of stress and strain were more uniform in the refined model of trabecular microstructure, in which stress and strain were mainly concentrated in trabecular bone. It was concluded that simulation of trabecular bone microstructure had a significant effect on the distribution of stress and strain at the implant-bone interface. These results suggest that trabecular structures could disperse stress and strain and serve as load buffers.

## 1. Introduction

Osseointegrated dental implants have been increasingly used to restore masticatory function in edentulous and partially edentulous situations and when only a single tooth is missing. Due to the absence of a periodontal ligament, osseointegrated implants, unlike natural teeth, react biomechanically in a different fashion to occlusal force. It is therefore believed that dental implants may be more prone to occlusal overloading, which is often regarded as one of the potential causes of peri-implant-bone loss and failure of the implant.

Finite element (FE) models have been developed in the past to quantify stress and strain fields in the bony tissue around dental implants [1]. As a numerical method for structure analysis that is suitable for complex biological structures, FE analysis has been widely used to evaluate the effect of various parameters (e.g., implant geometry, prosthesis design, and stress and strain distribution), in the peri-implant region [2–7].

In most reported studies, the tissues were assumed to be either homogeneous or nonhomogeneous and isotropic or anisotropic. Since the complex cancellous pattern was very difficult to determine, the cancellous bone network was ignored in these FEA studies. Therefore, it was always assumed that cancellous bone had a nonporous structure inside the inner cortical bone shell. Since trabecular bone architecture and density can vary greatly among individuals and between anatomical locations within the same individual, it is difficult to predict failure of a biomechanical etiology from these analyses using simplified models; therefore, relationships between load arising from the implant and actual structure of the surrounding cancellous bone have not been examined [8].

This limitation is due in part to the low resolution of CT scan images commonly used for finite element modeling. Even when using high-resolution cone-beam computed tomography (CBCT) in dentistry, the resolving power is around 0.2 mm/voxel, which is not enough to delineate trabecular structures. Recent studies have suggested that the trabecular structure of cancellous bone is closely related to bone strength [8]. Therefore, it is necessary to analyze dental biomechanics using a refined model in which the trabecular structure is simulated accurately to clarify the supporting function of the peri-implant bone.

With the development of microcomputed tomography and the improved performance of analytical systems, it is now possible to conduct biomechanical analysis, taking into consideration the actual morphology and structure of cancellous bone [8, 9]. Several studies have directly converted micro-CT scans of trabecular bone samples into 8-node hexahedral elements [10–13]. These voxel-based finite element models account naturally for bone morphology but result in large values for degrees of freedom, requiring extensive computing resources. Additionally, the model surfaces have jagged edges/aliasing, which may affect modeling quality, for example, at implant-bony tissue interfaces.

To analyze the strength of peri-implant jawbone, biomechanical investigations based on the trabecular structure of cancellous bone are necessary. Stegaroiu et al. compared the effect of loads from a simplified implant on the mandible when the trabecular structure of cancellous bone was simplified as a block and the actual structure was analyzed [14]. They reported that, in the actual trabecular structure, stress was dispersed over a wide area. Wolff et al. further assessed the effect of 4 different implant geometries on the strain distributions in the loaded bone leading to bone loss [15]. For these studies, some cadaveric mandibular bone segments without implants were scanned by micro-CT to obtain a geometrical model of the bone; subsequently, independent implant models were assembled into the bone structure using Boolean operations.

It should, however, be pointed out that bone tissue is a self-optimizing structure that adapts to exogenous load conditions; any difference between osseoresorption and osseoproduction leads to an increase or decrease in bone mass [16]. Inserting an implant into an edentulous ridge modifies the local stress state under a load and induces adaptive remodeling phenomena, affecting the morphological adaptation of the bone tissue, especially the trabecular architecture around the implant-bone interface. For this reason, to attain actual morphology and structure of jawbone with osseointegrated implants, the micro-CT scan acquisition should be performed on real implanted jawbone [17].

We also noticed that, to compare a microstructure model with the traditional macrostructure model, the trabecular structure of cancellous bone was filled as a block from the micro-CT scan data in previous studies [14, 18]. Nevertheless, the macrostructure model generated in this way is different from the traditional macrostructure model directly built from a conventional CT. We believe that it is more meaningful to obtain both micro-CT and conventional CT scans on the same jawbone sample to build comparing models, which should be more useful in the correct understanding of results from previous related studies.

The objective of this study was to assess the stress and strain magnitude and distribution within the 3D trabecular bone structure around osseointegrated dental implants. To describe precisely the geometry of anatomic parts, the inner morphology, and the implant-bony tissue interface conditions, we created individualized (animal-specific) finite element models of implanted mandible of the beagle dog, using both micro-CT scan and conventional CBCT scan.

## 2. Materials and Methods

### 2.1. Preparation of Animals

One 18-month-old male adult beagle dog (body weight 14 kg) with no lost teeth or occlusion abnormalities was used 1 week after introduction into the experiment. Preoperative and postoperative care were supervised by the university veterinary surgeon to ensure proper and humane treatment. The study protocol was reviewed and approved by Wenzhou Medical University Animal Research Committee.

### 2.2. Surgical Operations

**Figure 1:**
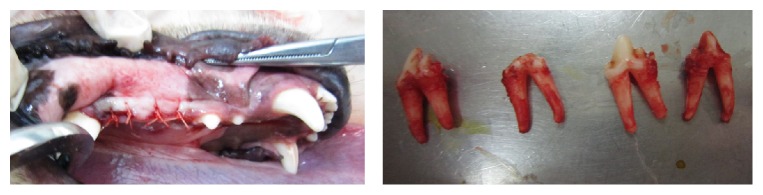
Extraction of the bilateral mandibular PM3 and PM4.

General anesthesia was induced by intramuscular administration of 10% ketamine hydrochloride (Gutian Pharmaceutical, Fujian, China) at 8–10 mg/kg and Sumianxin injection II (Shenda Animal Pharmaceutical, Jilin, China) at 0.1 mL/kg. Local infiltration anesthesia with 2% lidocaine (Chengyi Pharmaceutical, Zhejiang, China) was also used at the site of tooth extraction. Disinfection around the mouth and oral cavity was performed with 0.5% chlorhexidine (Nanyue Pharmaceutical, Shenzhen, China) before operation. Bilateral mandibular third and fourth premolars (PM3 and PM4) were extracted from each dog using a minimally invasive operation. Following tooth extraction, the extraction wounds were approximated and closed with 4/0 polyglactin 910 (Vicryl, Ethicon) absorbable sutures (Figure 1).

After a 3-month healing period, proper alveolar bone healing was confirmed by spiral CT and two dental implants were surgically inserted in the bilateral mandibular premolar region. A midcrestal incision was made on the healed alveolar ridge to expose the respective sites. The implant sites were prepared using pilot drills of diameters 2.2 mm, 2.8 mm, and 3.5 mm, in that order, under irrigation with sterile physiological saline solution. Regular neck-type SLA implants (Straumann® System, Basel, Switzerland) with a length of 8 mm and a diameter of 4.1 mm were placed. After the implants had been placed in this submerged fashion, the flaps were closed with a simple interrupted technique using 4/0 polyglactin 910 (Vicryl, Ethicon), and primary soft tissue closure was achieved with no additional procedures. The dog was euthanized 3 months later, and the mandible was resected. A wire saw was used to cut the specimen into separate bony blocks containing 2 implants each.

### 2.3. Micro-CT and CBCT Scan

**Figure 2:**
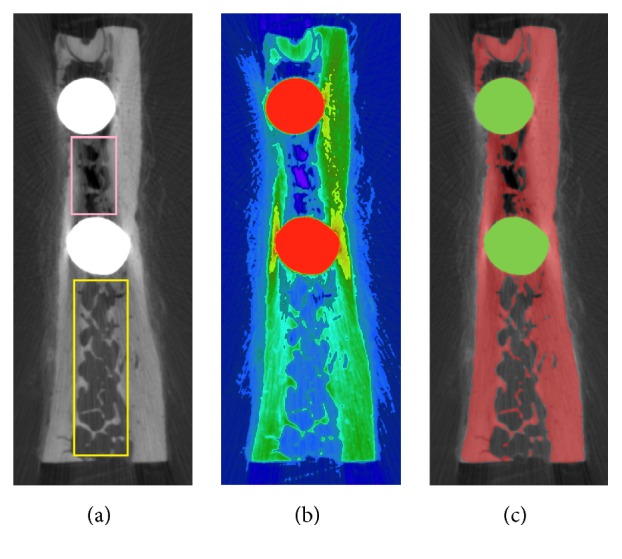
Process of micro-CT image. (a) One slice of micro-CT image. (b) Pseudocolor mapping image. (c) Image segmentation result.

Micro-CT images of specimens were taken using a micro-CT scanner (*μ*CT80 micro-CT, Scanco Medical, Switzerland) at the spine and spinal cord injury research laboratory at the affiliated Nanfang Hospital, Southern Medical University, China. All samples were scanned at an energy of 70 kV and an intensity of 114 *μ*A with a scanning thickness of 18 *μ*m. The scanning time used for each sample was approximately 6 h with a resolution of 2048 × 2048. The cancellous bone structure showed rod-like trabeculae with round voids between them as shown in Figure 2(a). Altogether, 1500 two-dimensional slice images were obtained, and the raw data were saved in DICOM format. In addition, CBCT images of specimens were taken using a Newtom cone-beam CT with a scanning thickness of 0.25 mm. Altogether, 320 two-dimensional slice images were obtained and saved in DICOM format. All scan images were imported to the self-developed medical image processing and biomechanical modeling system E-3D software (http://www.e-feature.net/), which stacks the images for visualization and segmentation based upon gray-scale density corresponding to various degrees of mineralization.

### 2.4. Refined Mandible Model with Trabecular Microstructure

Micro-CT images were first subjected to noise elimination. Binarization was subsequently performed using thresholds obtained by discrimination analysis. Nevertheless, the result of binarization was not perfect due to imaging artefacts caused by the presence of implant metal in the mandible.

This problem can be clearly demonstrated in Figure 2. While observing the gray-level micro-CT image in Figure 2(a), human eyes can easily identify the cancellous bone: the local luminance of the trabecular structure region between the 2 implants (indicated by a pink rectangle), though smaller than that of the trabecular region outside the implants (indicated by a yellow rectangle), should still be larger than that of the background area inside the yellow rectangle. Applying a pseudocolor mapping method to generate a color image from the gray image as shown in Figure 2(b) reveals that the new color of the trabecular structure between the 2 implants is very similar to that of the background area inside the yellow rectangle. Because of the similar gray-level pixel values in the micro-CT image, it is difficult to achieve a perfect result with threshold binarization.

To address this problem, we developed some interactive image segmentation tools in the E-3D software, mainly based on graph cuts technology [19]. Under the guidance of the pseudocolor image, the user can improve the threshold segmentation result with a few interactions to accurately extract local trabecular structure.  Figure 2(c) illustrates the final segmentation results, with the bone region covered by a red mask and the implant region covered by a green mask.

**Figure 3:**
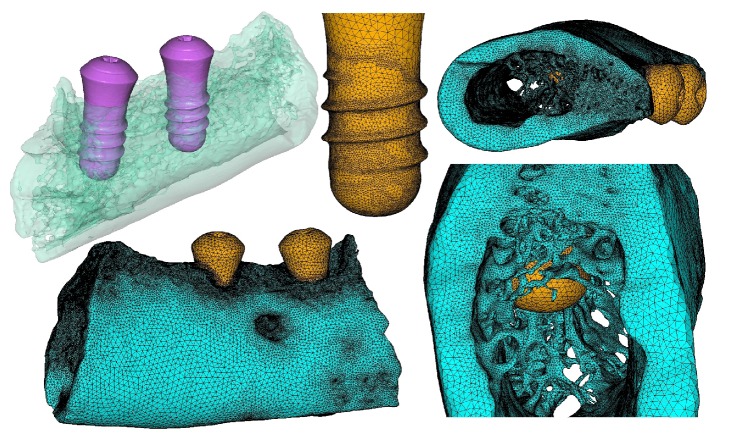
Trabecular microstructure mandible model containing 2 osseointegrated implants.

3D geometry models of mandible bone tissue (including the trabecular microstructure) and two implants were subsequently reconstructed from segmentation results. For the implants, finite element mesh models can be directly discretized by 10-node quadratic tetrahedral elements from the geometry solid models using an adaptive meshing tool, creating biomechanically significant regions, such as implant threads with finer element sizes, to enhance the accuracy of simulation results, as shown in the middle upper part of Figure 3. For the bone tissue model, because it contains a large amount of trabecular microstructure, the geometry solid model may have some topology errors and undesired geometric features, which should be preprocessed with some geometry repair and optimization operations. The final trabecular microstructure mandible model can then be generated using the adaptive meshing tool, as shown in low right part of Figure 3, which contains 2150476 elements and 3303031 nodes. One implant model contains 111833 elements and 170402 nodes; the other implant model contains 107795 elements and 164426 nodes. For the bone-implant interface between the mandible and implants assumed to be complete or to have 100% osseointegration, contact area was modeled as a continuous bond.

The material properties of bone tissue and the dental implants were defined using a homogeneous isotropic linearly elastic material model, explicitly described by two parameters: Young's modulus (*E*) and Poisson's ratio (*ν*). For titanium implant these were defined as *E* = 110 GPa and *ν* = 0.35 [8, 14]. The trabecular and cortical bone material characteristics were assumed to be similar at the microlevel in keeping with the literature [15]. As such, similar material properties were used for both bone tissues, specifically *E* = 14.4 GPa and *ν* = 0.309 [14, 15].

### 2.5. Simplified Macrostructure Mandible Model

**Figure 4:**
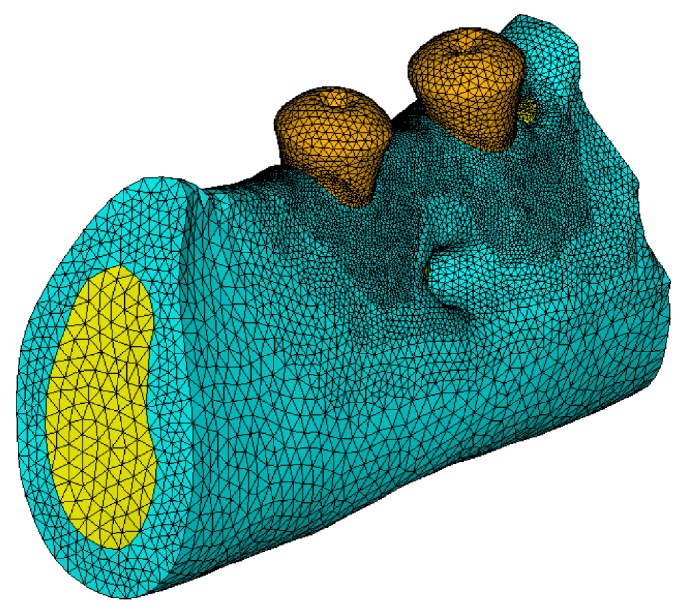
Simplified macrostructure mandible model containing osseointegrated implants.

CBCT images were also subjected to noise elimination. As there is no need to extract trabecular microstructure, the E-3D software can automatically distinguish the cortical and cancellous bone regions. However, due to lower scanning accuracy, the implant boundaries in CBCT were not very clear, and geometrical features such as implant threads were completely lost. To address this problem, the implant models reconstructed from the micro-CT scan were registered to the CBCT data space. Boolean operations were then performed to assemble accurate implant models and the CBCT cortical and cancellous bone models, which were discretized using the adaptive meshing tool in E-3D software, as shown in Figure 4. The final macrostructure mandible model contains 2150476 elements and 3303031 nodes. One implant model contains 111833 elements and 170402 nodes; the other implant model contains 107795 elements and 164426 nodes.

Using previously reported values of mechanical properties as reference, Young's moduli of 110, 14.4, and 0.48 GPa and Poisson's ratios of 0.35, 0.309, and 0.225 were set for the titanium implant, cortical bone, and cancellous bone, respectively [13].

### 2.6. Boundary and Loading Conditions

All nodes at the mesial and distal borders of the mandibular bone segment were fixed in all directions to represent continuity within the mandible [15, 17], and a vertical load of 50 N (parallel to the long axis of the implant) was applied to the top of the implants in each mandible model.

## 3. Results

The stress and strain magnitude and distribution within the bone tissue around osseointegrated dental implants were analyzed.

### 3.1. Stress Variation on Implant-Bone Interface

**Figure 5:**
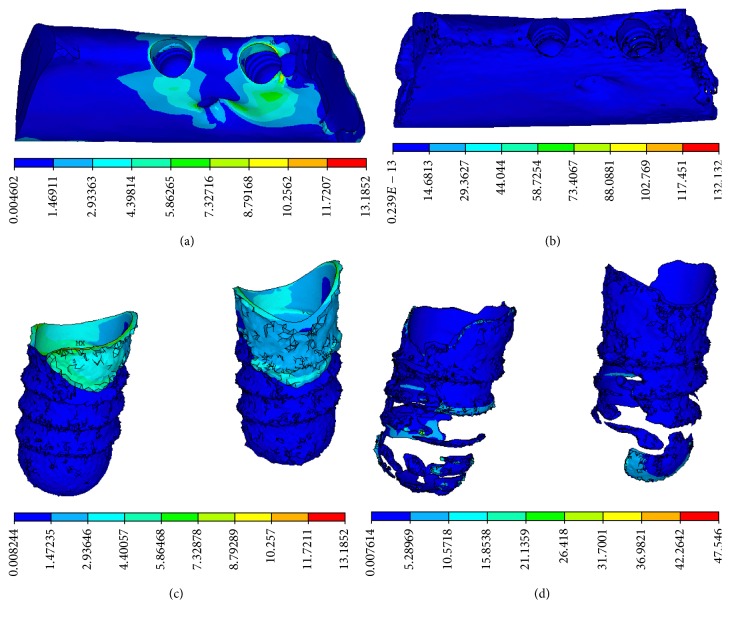
Equivalent stress distributions. (a) CBCT model and (b) micro-CT model on the vertical view of the alveolar bone around the implants. (c) CBCT model and (d) micro-CT model on the lingual view of the alveolar bone around the implants.

Equivalent stress concentration at the implant-bone interface was obvious in a CBCT model, in which stress concentration appeared in the neck of the implant-bone interface, characterized by a cortical shell. The distribution of equivalent stress was uniform in a micro-CT model, in which stress concentration appeared in the lower region of the implant-bone interface, characterized by trabecular bone. It was apparent that stress concentration was more obvious in the CBCT model than those in the micro-CT model, as shown in Figure 5.

**Figure 6:**
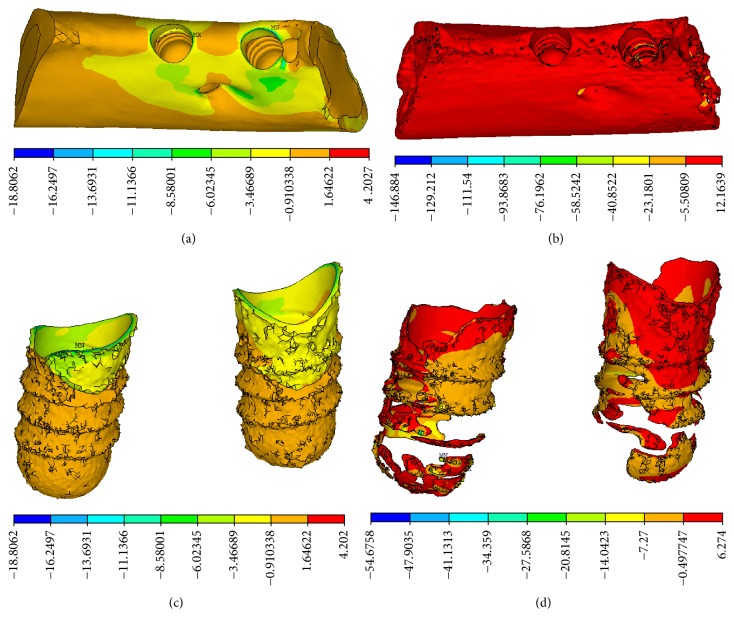
Compressive stress distributions. (a) CBCT model and (b) micro-CT model on the vertical view of the alveolar bone around the implants, (c) CBCT model, and (d) micro-CT model on the lingual view of the alveolar bone around the implants.

In the CBCT model, compressive stress concentration appeared at the neck of implant-bone interface, characterized by cortical bone. In the micro-CT model, the distribution of compressive stress was dispersed widely and mainly appeared in the lower region of the implant-bone interface, characterized by trabecular bone on the threaded position, as shown in Figure 6.

**Figure 7:**
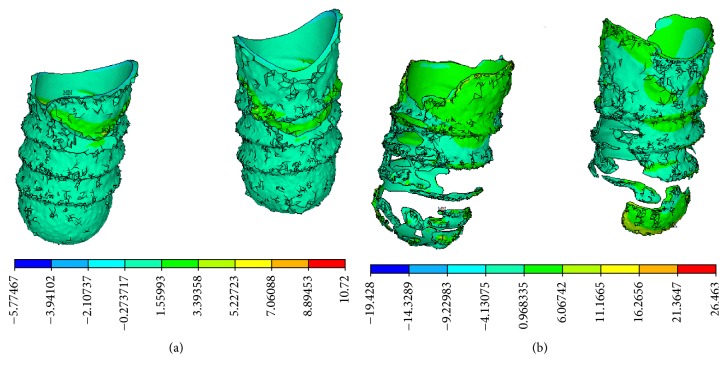
Tensile stress distributions on the lingual view of the alveolar bone around implants. (a) CBCT model. (b) Micro-CT model.

In the CBCT model, tensile stress appeared concentrated in the upper portion of implant-bone interface, characterized by the junction of cortical bone and cancellous bone. In the micro-CT model, tensile stress was dispersed widely and appeared mainly at the neck of cortical bone and in the bottom of trabecular bone, as shown in Figure 7.

**Figure 8:**
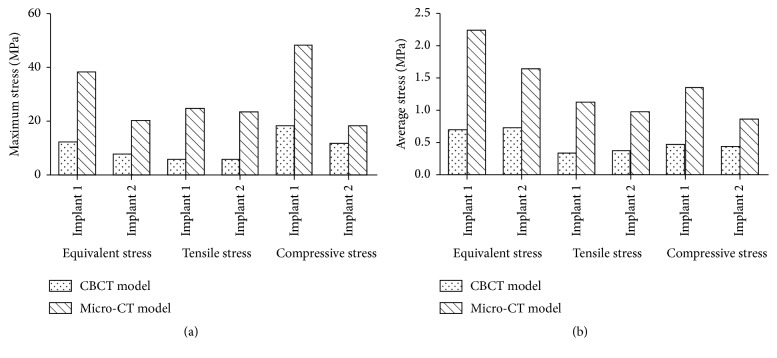
Maximum stress (a) and average stress (b) of implant-bone interface.

The maximum and average values of stress at the implant-bone interface in the micro-CT model increased remarkably, compared with those in CBCT model, as shown in Figure 8. The maximum values of equivalent stress, tensile stress, and compressive stress on the bone around implant 1 increased by 214%, 330%, and 165% in the micro-CT model over those in the CBCT model, while the average values for the same parameters increased by 219%, 239%, and 187% in micro-CT model. The maximum values of equivalent stress, tensile stress, and compressive stress on the bone around implant 2 increased by 161%, 296%, and 59% in the micro-CT model over those in CBCT model, while the average values of the same parameters increased by 125%, 153%, and 95% in the micro-CT model.

### 3.2. Strain Variation on Implant-Bone Interface

**Figure 9:**
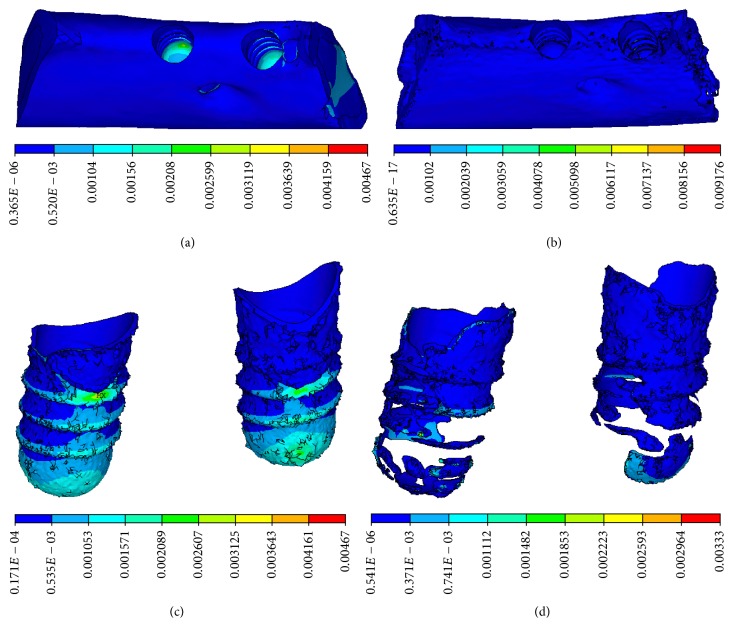
Equivalent strain distributions. (a) CBCT model and (b) micro-CT model on the vertical view of the alveolar bone around the implants. (c) CBCT model and (d) micro-CT model on the lingual view of the alveolar bone around the implants.

**Figure 10:**
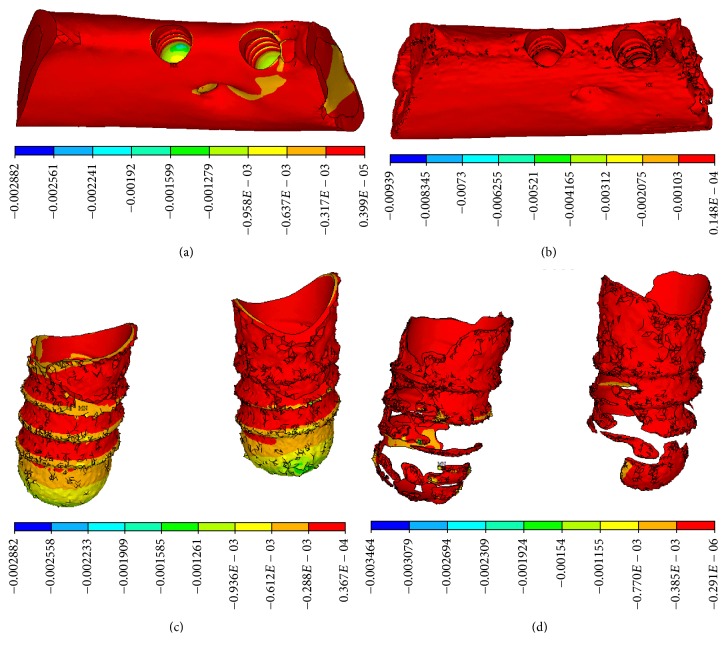
Compressive strain distributions. (a) CBCT model and (b) micro-CT model on the vertical view of the alveolar bone around the implants. (c) CBCT model and (d) micro-CT model on the lingual view of the alveolar bone around the implants.

Equivalent strain and compressive strain concentration at the implant-bone interface were obvious in the CBCT model, in which strain concentration appeared on the threaded position and at the bottom of cancellous bone, as shown in Figures 9 and 10. The distributions of equivalent strain and compressive strain were uniform in the micro-CT model, in which stress concentration appeared in the lower region of the implant-bone interface, characterized by trabecular bone.

**Figure 11:**
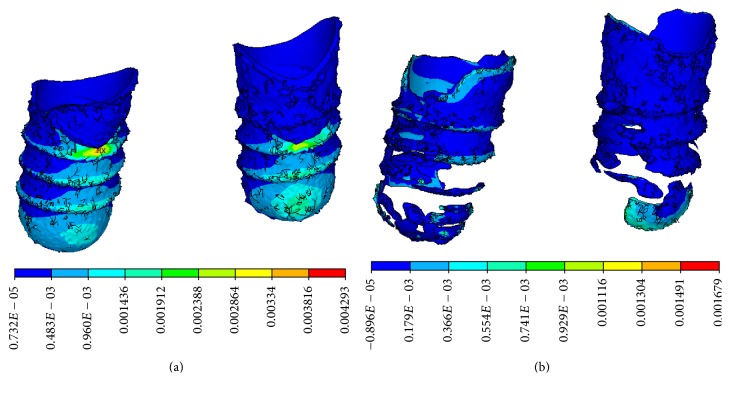
Tensile strain distributions on the lingual view of the alveolar bone around the implants. (a) CBCT model. (b) Micro-CT model.

In the CBCT model, tensile strain concentration at the implant-bone interface was obvious and appeared on the threaded position and at the bottom of cancellous bone. In the micro-CT model, the tensile strain was dispersed widely and mainly appeared at the neck of cortical bone and in the bottom of trabecular bone, characterized by trabecular bone, as shown in Figure 11. It was apparent that strain concentration was more obvious in the CBCT model, compared with that of the micro-CT model.

**Figure 12:**
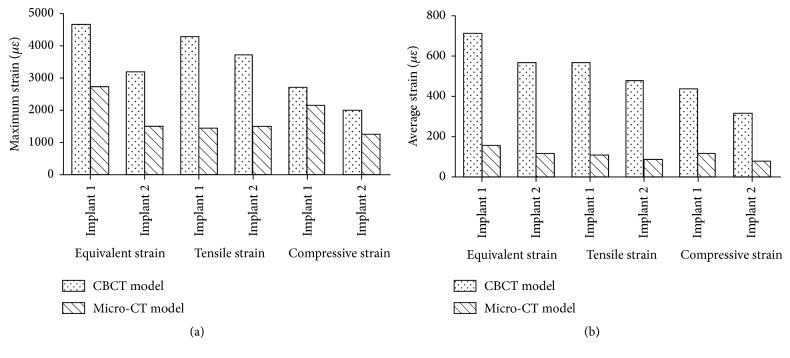
Maximum strain (a) and average strain (b) at the implant-bone interface.

The maximum and average values of strain at the implant-bone interface in the micro-CT model decreased remarkably compared with those in the CBCT model, as shown in Figure 12. The maximum values of equivalent strain, tensile strain, and compressive strain on the bone around implant 1 decreased by 42%, 67%, and 21% in the micro-CT model compared with those in the CBCT model, while the average values for the same parameters decreased by 78%, 81%, and 74% in the micro-CT model, respectively. The maximum values of equivalent strain, tensile strain, and compressive strain on the bone around implant 2 decreased by 53%, 60%, and 38% in the micro-CT model compared with those in the CBCT model, while the average values for the same parameters decreased by 80%, 82%, and 75% in the micro-CT model, respectively.

## 4. Discussion

In previous studies, the main difficulties in modeling the implant-bone complex in the dental area arose when trying to precisely describe the geometry of anatomic parts, the inner morphology and material properties of bony tissues, the true 3D loading and boundary conditions, and the nature of implant-bony tissue interfaces. Most standard methods to predict bone quality are based solely on apparent density measurements. However, apparent density alone neither explains all variation in mechanical properties nor accounts for the structural anisotropy of trabecular bone. Therefore, apparent density may not be sufficient in itself to accurately predict bone quality. Ulrich et al. have suggested that micro-CT-based FEA provides additional information about anisotropy and mechanical properties in a direct and nondestructive way [20].

Microcomputed tomography (*μ*CT) is an emerging technique for the nondestructive assessment and analysis of three-dimensional trabecular bone architecture. Nondestructive *μ*CT measurements permit not only quantitative bone morphometry but also the assessment of other important microstructural features in the determination of the mechanical integrity of trabecular bone [21]. *μ*CT-based finite element (*μ*FE) analysis is a widely used tool to quantify stresses and strains in bone. Specifically, the use of voxel-based FE models allows for a highly accurate representation of bone external geometry as well as the internal microarchitecture of bone [22–24].

Recent papers on bone biomechanics have discussed the need to consider trabecular bone architecture. Verhulp et al. [25] reported that stresses were dispersed by trabecular bone at the proximal head of the femur. Homminga et al. [26] suggested that a strong relationship existed between trabecular bone architecture and bone strength. Micro-CT, as applied in the present study, has become a well-established tool in the field of bone mechanics, where it is predominantly used to reconstruct the structure of trabecular bone, and has recently been employed in investigations of peri-implant bone [27–29].

The modeling process has a large influence on the results of an FE analysis. It is not possible to make predictions without proper descriptions of morphologic and geometric aspects. Nevertheless, most previous FE models of bone with dental implants were generated by approximating the cancellous bone to a regular geometrical block shape [1]. In stark contrast with previous studies of implant-bone interactions found in the literature, strains obtained from the FE analyses are given at the trabecular level, thus providing a more realistic approach than continuum models, which consider the peri-implant bone as a geometrically homogeneous continuum medium. The additional advantage of modeling explicitly the trabecular microstructure of bone instead of assuming a representative homogenized continuum volume, whereby one assigns anisotropic mechanical properties, is that anisotropy is naturally accounted for by means of structural properties [12].

In this study, the maximum and average values of stress increased remarkably over those in the CBCT model. The maximum equivalent stresses at the implant-bone interface in the micro-CT model were 2.6-fold and 3.1-fold higher than those in the CBCT model, similar to the results of Stegaroiu et al. [14]. In addition to remarkable differences between the respective stress and strain values, stress distributions were distinctly different between the two models. In the CBCT model, high stress was concentrated mainly in the neck region of the implant-bone interface, characterized by cortical shell. In the micro-CT model, stress was distributed over wide areas at the implant-bone interface and concentration appeared in the lower region of the implant-bone interface, characterized by trabecular bone. Although remarkable differences were found between the stress distributions in the two models, strain distributions were similar, showing concentration of strain around the threaded position of cancellous bone and trabecular bone. Although the implant was loaded along its long axis by a vertical force, the maximum deformations of the trabecular structure were reached at the periphery of the implant above its bottom base.

Higher trabecular bone stress around the implant-bone interface of the micro-CT model can be explained by a decrease of the bone substance as compared with the CBCT model. The porous bone structure also allowed a greater displacement of the implant, which triggered a greater deformation of the cortical bone and thus a higher cortical bone stress [14]. In contrast to the CBCT model, the distribution patterns and higher stresses in the micro-CT model may explain why trabecular bone areas supporting load transfer from implants undergo remodeling. These results implied that trabeculae disperse the load and transform themselves into a shock-absorbing structure [9].

Stegaroiu et al. compared the effect of stress with FEA when the trabecular structure of cancellous bone was simplified as a block and to that when the actual structure was analyzed. They reported that stress was dispersed over a wide area in the actual trabecular structure [14]. Matsunaga et al. reported a large concentration of stress in surrounding trabecular bone due to load transfer from the implant to the mandible using a micro-CT model that considered the actual cancellous bone structure of the mandible with an embedded implant [12]. These results were all similar to ours. Previous studies showed that cortical bone around implants dispersed stress. Our studies showed that trabeculae of cancellous bone at the implant-bone interface dispersed stress by forming load transfer paths. This suggests that not only cortical bone but also cancellous bone plays a major role in supporting the functional pressure exerted via the implant [8].

The heterogeneity of the bone tissue at the microscale was not considered in this study, since the material is assumed to be homogeneous for each model, according to the procedures described in the literature. In the trabecular region, the thickness of the trabeculae and their distance could result in heterogeneities having a scale size comparable to that of threads. Further consideration pertaining to the state-of-the-art of modeling of the postelastic behavior of bone trabecular tissue is warranted [30].

## 5. Conclusions

This finite element study revealed that the stress at the implant-bone interface in micro-CT model increased remarkably over that in CBCT model, while the strain behaved in the opposite manner. However, the distribution of stress and strain was more uniform in the micro-CT model than in the CBCT model. These variations were indeed found to have a significant impact on the distribution patterns of stress and strain at the implant-bone interface when the trabecular bone microstructure was simulated. These results suggest that trabecular structures could disperse stress and strain and serve as load buffers.
